# Proof-of-concept study of the TriBot: a robot-based test setup for biotribological analyses of curved articular surfaces

**DOI:** 10.3389/fbioe.2025.1546060

**Published:** 2025-04-25

**Authors:** Luisa de Roy, Moritz Roderigo, Jonas Schwer, Klaus Schlickenrieder, Anita Ignatius, Andreas Martin Seitz

**Affiliations:** ^1^ Institute of Orthopedic Research and Biomechanics, Center for Trauma Research, Ulm University Medical Center Ulm, Ulm, Germany; ^2^ Ulm University of Applied Sciences, Faculty of Production Engineering and Management, Ulm, Germany

**Keywords:** friction, articular cartilage, robotic, tribometer, tissue integrity, osteoarthritis

## Abstract

**Introduction:**

Investigations on the articular cartilage (AC) frictional properties contribute to a better understanding of knee joint functionality. We identified the need for a tribological setup that allows for friction measurements on curved AC surfaces, without disrupting its structural integrity, under orthogonal contact conditions and controlled normal force application. Therefore, a robotic-based tribometer–the TriBot–was developed and validated in a two-part proof-of-concept study.

**Methods:**

First, the friction coefficients of polyoxymethylene pins on three different polyamide (PA) shapes were determined for validation purposes. Second, the frictional properties on porcine tibial plateaus were investigated. Trajectories on the medial and the lateral tibial surface were tested in the intact cartilage state and after inducing an anteromedial local defect.

**Results:**

No significant differences in the friction coefficients of the PA samples were found. Inducing an anteromedial cartilage defect significantly increased friction on the affected trajectories (+30%, p < 0.05).

**Discussion:**

Our findings showed that the robotic tribometer is suitable for friction measurements on complexly shaped samples and that the system can detect differences in cartilage friction due to structural tissue damage. Overall, the robotic tribometer has the potential to advance our understanding of the knee joint’s friction-related functionality.

## 1 Introduction

Detailed knowledge of the frictional properties of the articular cartilage (AC) contributes to a better understanding of joint functionality in both, healthy and diseased conditions (Jay and Waller, 2014; Li et al., 2021). The greatest challenge for tribological investigations of the AC in the knee joint remains the complex, curved geometry of the tibial and femoral articulating surfaces. Numerous tribometers have been developed over recent decades to study AC friction at different levels of complexity (Link et al., 2020; Rajankunte Mahadeshwara et al., 2024). At the joint level, pendulum setups allow for the investigation of whole knee joint friction (Link et al., 2020; Crisco et al., 2007). While in passive pendulum tests a single friction coefficient for the entire joint is derived from the passive knee joint motion (Link et al., 2020; Crisco et al., 2007), active pendulum setups allow for direct measurement of frictional forces (McCann et al., 2009; Mederake et al., 2022). Although both setups offer a comprehensive view taking the geometry of the joint into account, pendulum setups lack the specificity to assess AC friction at different locations on the articulating surfaces. This is critical because AC friction can vary across the joint in response to the mechanical environment and due to local differences in surface topography (Espinosa et al., 2021; Desrochers et al., 2013). Moreover, location-specific frictional properties “have significant implications for our understanding of osteoarthritis”, a degenerative joint disease characterized by varying degrees of localized AC damage (Moore and Burris, 2015). At a tissue level, pin-on-plate tribometers are commonly used to investigate local AC frictional properties (Link et al., 2020). Here, an axial load is applied on a cylindrical sample (pin) while a flat sample (plate) is moved parallel to it, thereby generating a friction force (Link et al., 2020). The orthogonality between the samples allows for the calculation of a friction coefficient µ according to Coulomb’s law (µ = friction force/normal force). However, these pin-on-plate tribometers require the extraction of cylindrical and/or flat AC samples from the tissue composite (Warnecke et al., 2019; Warnecke et al., 2017). This disrupts the natural integrity of the AC, which is very likely to affect its frictional properties (de Roy et al., 2023). Recently, a modified pin-on-plate tribometer was introduced by Schütte et al., which allows the investigation of non-planar osteochondral plates (Schütte et al., 2020). While this system preserves cartilage integrity during friction testing, the normal force applied orthogonally to the AC surface varies with its curvature. The resulting variation in normal force may have implications for the friction coefficient, because cartilage friction is highly load dependent (Warnecke et al., 2019; Furmann et al., 2020; Gleghorn and Bonassar, 2008). To the best of our knowledge, there is no tribological setup that allows the determination of frictional properties on curved AC surfaces without disrupting its structural integrity under orthogonal contact conditions and controlled normal force application. Therefore, the aim of this study was to develop a novel method that allows the determination of the frictional properties at different locations on curved joint surfaces in accordance with Coulomb’s law. A newly developed tribometer with an industrial robot as an actuator–the TriBot–is demonstrated here in a two-part proof-of-concept study. For validation purposes, the friction coefficients of polyoxymethylene (POM) against polyamide (PA) were first quantified. Three PA samples with different shapes were investigated in the robotic tribometer. Because the material pairing was the same for all shapes, it was hypothesized that the assessed friction coefficients would not differ between the samples. In the second part of the study, the robotic tribometer was used to determine AC friction along trajectories at different anatomical regions on porcine tibial plateaus with a POM pin as the friction partner. The tests were performed on macroscopically intact medial and lateral tibial AC (intact) and after inducing a local anteromedial AC defect using sandpaper (defect). It was hypothesized that the mechanically induced cartilage damage results in increased friction on the affected trajectories.

## 2 Materials and methods

### 2.1 Robotic tribometer

#### 2.1.1 Design and technical description

**FIGURE 1:**
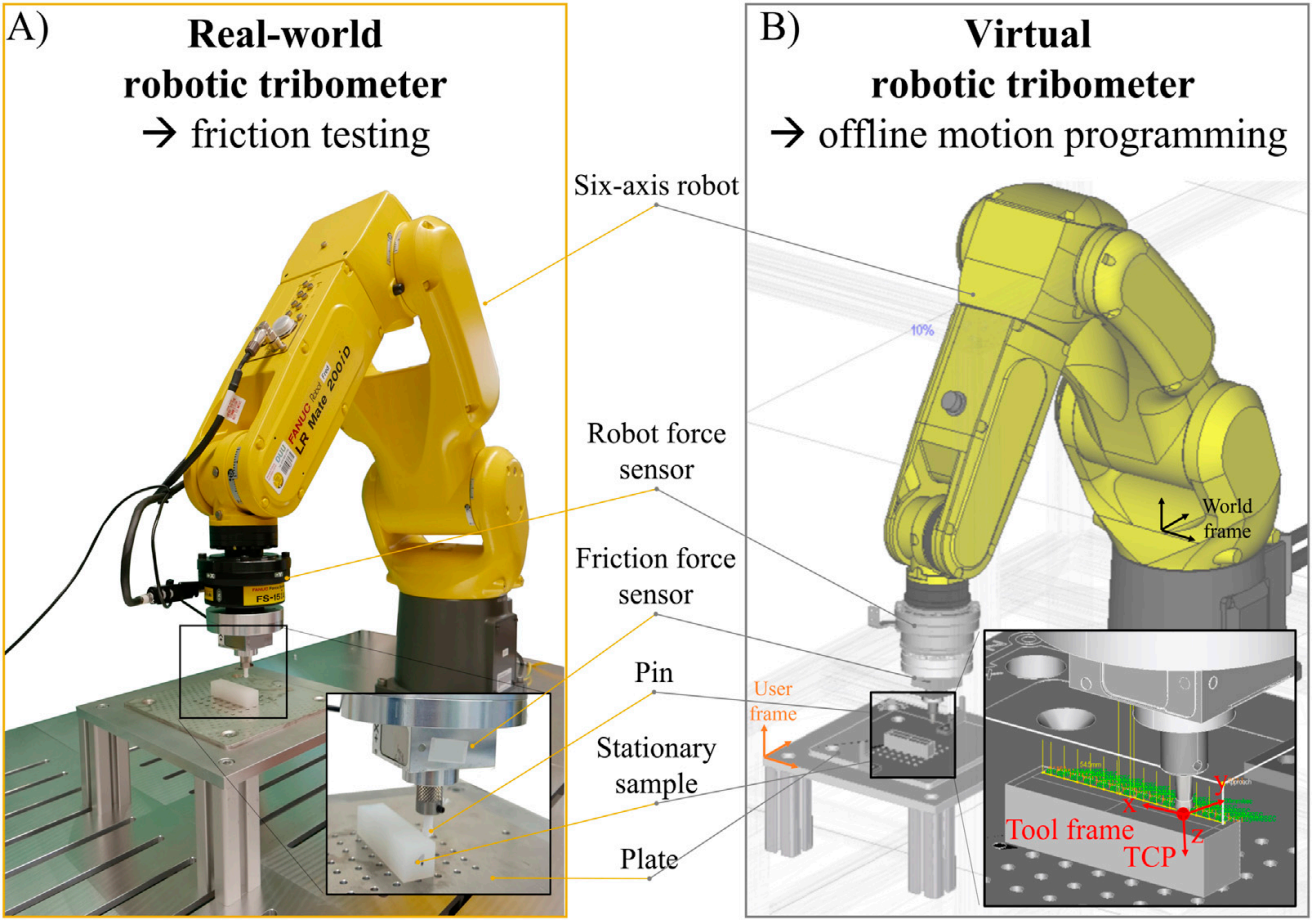
Robotic tribometer setup. **(A)** Real-world robotic tribometer: a six-axis industrial robot was used as actuator during friction testing, allowing the orthogonal positioning of a pin along the surface of a stationary sample mounted on a plate. The internal force sensor of the robot was used for load control, while the friction force was recorded with an additionally equipped high-precision force sensor. **(B)** Virtual robotic tribometer: a virtual representation of the robotic tribometer was created to perform offline motion programming in the robot’s simulation software. The position and orientation of the pin during testing was defined by a tool frame and tool center point (TCP).

The robotic tribometer design was developed based on the principle of a pin-on-setup. A six-axis industrial robot (FANUC LR Mate 200iD, controller: R-20iB Plus, FANUC Deutschland GmbH, Germany) was used as the actuator (Figure 1A). Its six degrees of freedom (DOF) allow a pin to be moved along trajectories on complex shapes, while facilitating an orthogonal positioning of the pin to the surface. The robot has a maximum load capacity of 70 N, a maximum reach of 717 mm (repeatability ±0.01 mm) and a maximum moving velocity of 750 mm/s. The robot was mounted on a ground plate inside a robotic workcell in accordance with ISO 10218. The robot flange is equipped with an integrated six-axis force sensor (robot force sensor) (FS-15 iA, maximum load F_x,y,z_ = 147 N, maximum moment M_x,y,z_ = 11.8 Nm, resolution: 0.39 N; 0.04 Nm, FANUC Deutschland GmbH) which was used for force-controlled movements during testing. Additionally, a high-resolution three-axis force sensor (friction force sensor) (K3D40 ME, 3 DOF, maximum F_x,y_ = 20 N, maximum F_z_ = 50 N, resolution: 0.005 N, ME-Messsysteme GmbH, Germany) was mounted on top of the six-axis sensor to record the processing forces (friction and normal force). The moving sample (pin) was fixed in a sample holder mounted on top of the friction force sensor. The other, stationary friction contact partner (stationary sample) was mounted on a plate within the workcell. The robot motion and normal force control were performed by the robot controller, whereby the position data of the pin and the force data of the robot sensor were recorded. Force data acquisition of the three-axis sensor was performed using a custom-made LabVIEW program (LabVIEW 2019; National Instruments, United States). All data were recorded with a sampling rate of 100 Hz.

#### 2.1.2 Robot motion programming

**FIGURE 2:**
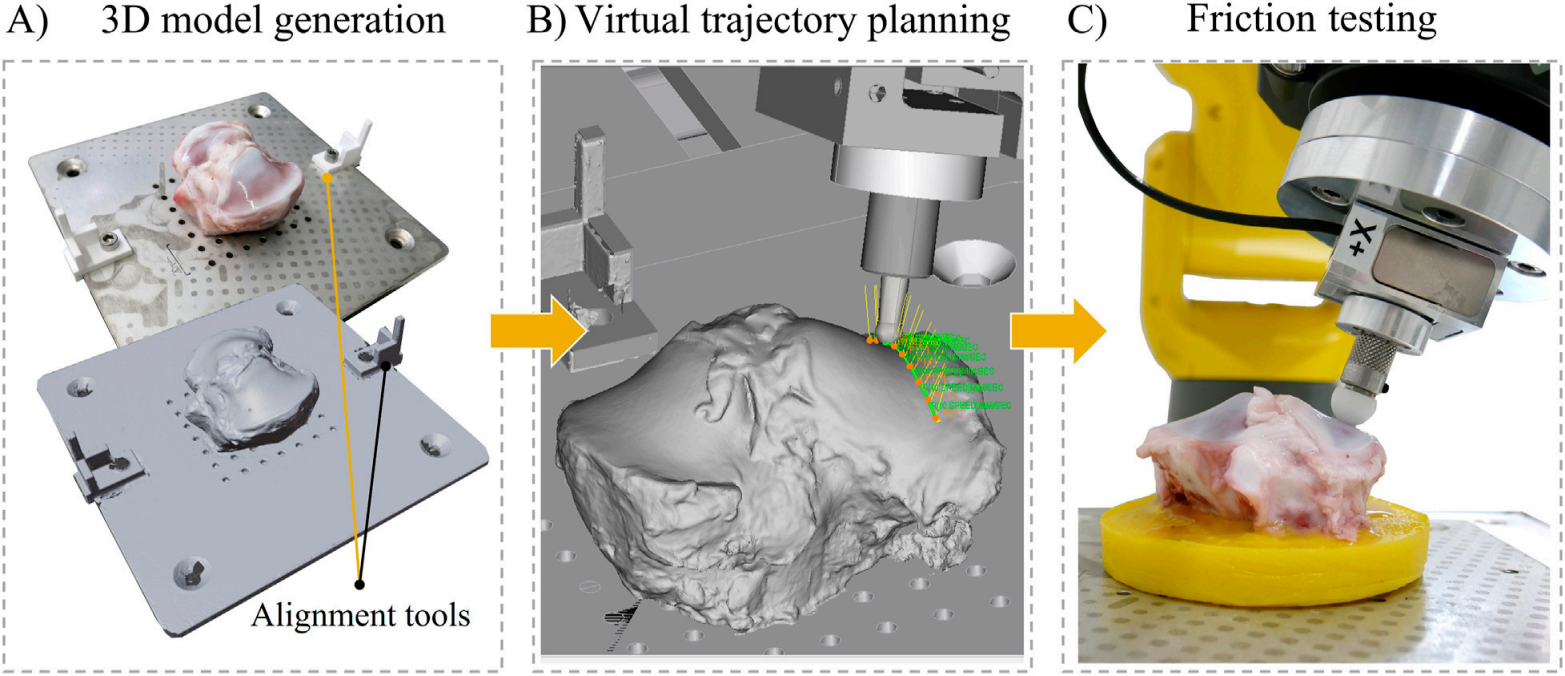
Workflow of friction tests conducted with the robotic tribometer on the example of a porcine tibial plateau as a stationary sample. **(A)** A three-dimensional (3D) model of the tibial plateau was generated using a 3D scanner for the virtual trajectory planning and robot motion programming in the system’s simulation software. Alignment tools enabled a precise positioning of the tibial plateau in the virtual tribometer. **(B)** Trajectories were defined by setting target points on the surface of the 3D model. **(C)** The generated robot motion program was transferred to the robot controller to perform the friction tests in the real-world tribometer.

A virtual representation of the tribometer was created using the offline three-dimensional (3D) robot simulation software (FANUC ROBOGUIDE, version 9, FANUC Deutschland GmbH) (Figure 1B). This software enabled the graphical programming of robot motions based on 3D models of a pin and stationary sample. The pin’s orientation and position were defined by a tool coordinate system (aligned with the axis of both force sensors) and tool center point (TCP) located at the tip of the pin (Figure 1B). Trajectories were defined offline by setting target points with a fixed distance of 2 mm on the surface of the 3D model of the stationary sample (linear movements with 100% rounding accuracy between points) (Figures 2A, B). These were subsequently discretized into Cartesian coordinates. This Cartesian information was used by the robot to move the TCP, hence the tip of the pin, along the trajectory during testing (motion type: linear, termination type: continuous). Thereby, the robot aligned the pin’s x-axis (which was coincident with the x-axis of both force sensors) along the trajectory in the direction of motion. In this way, the x-force recorded corresponded to the friction force used for the calculation of the friction coefficient. To simultaneously adjust the pin orthogonally to the stationary sample’s surface, the z-axis of the pin (coincident with the z-axis of both force sensors) was aligned collinear to the normal vectors, being directed from the surface normal at the target points. Constant normal force application along the z-axis of the pin was performed with the robot’s contouring application (Contouring Function, FANUC Deutschland GmbH). After entering the velocity at which the robot moves along the trajectory, the robot motion programming was completed. Finally, the robot’s motion program was generated by the software, transferred to the robot controller and the friction experiments could be performed.

### 2.2 Proof-of-concept experiments

#### 2.2.1 POM on PA

**FIGURE 3:**
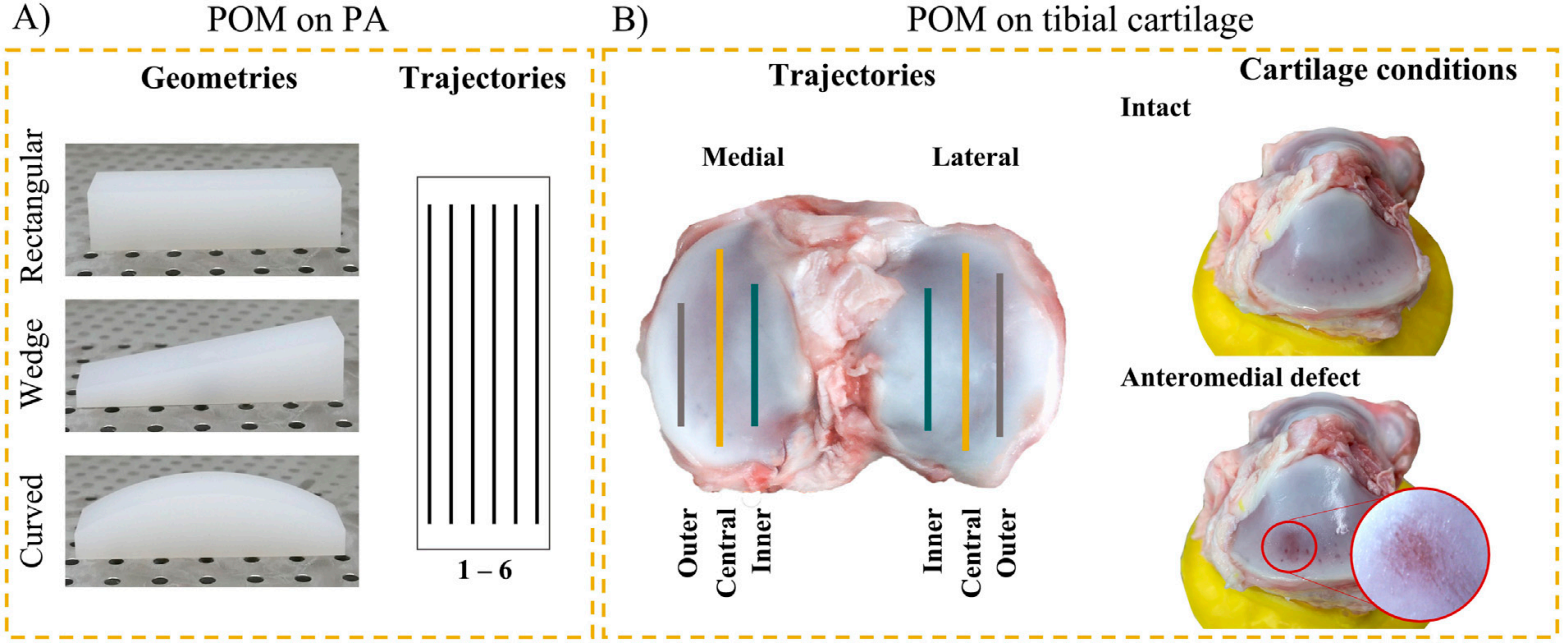
Proof-of-concept experiments: **(A)** The friction coefficients of POM (pin) on three PA shapes (stationary sample) were determined. Six trajectories were investigated on each geometry. **(B)** The friction coefficients of POM on tibial plateaus were assessed. Two articular cartilage conditions were tested: initially in the intact condition and after induction of an anteromedial articular cartilage defect.

For validation purposes, the friction coefficients of POM against PA under dry sliding conditions were determined (Figure 3A). This tribological system was chosen because it is characterized by friction coefficients in the range of those reported for AC (Rajankunte Mahadeshwara et al., 2024; Miler et al., 2019). A hemispherical geometry (Ø 12.7 mm) made of POM (HOSTAFORM C13021) (Schütte et al., 2020) was used as a pin and fixed in the robot. Three different stationary sample shapes were designed using a computer-aided design (CAD) software (Inventor Professional 2025; Autodesk GmbH, Germany) and manufactured of polished PA6-E: a rectangular sample, a wedge-shaped sample with an inclination angle of 10° (wedge) and a convex shaped (curved) sample (Figure 3A). The workflow and friction testing protocol were as follows: In the virtual tribometer, six parallel trajectories, spaced 2 mm apart, were defined on the surface of the PA sample’s 3D model, which was readily available from the design process using the CAD program. The stroke length of each trajectory was 50 mm. Before testing, the PA sample and POM pin were cleaned in an ultrasonic bath with ultrasonic cleaner (Ultrasanol 7, Carl Roth GmbH + Co. KG, Karlsruhe, Germany) for 5 min and dried in ambient air similarly for 5 min (Schütte et al., 2020). The PA sample was subsequently fixed on the plate with two threaded screws. During testing, a constant normal force of 5 N and a constant velocity of 1 mm/s were applied. These test conditions were employed in the system’s validation experiments because they were defined as physiologically relevant for the experiments on tibial cartilage. The test conditions were applied for five reciprocal cycles on each trajectory. Between the reciprocal test runs, both the sample and the pin were cleaned in an ultrasonic bath as described above to remove any possible abrasive particles from the test. A predefined hole pattern on the sample plate allowed the re-fixation of the PA sample at the exact same position. All tests were performed on the same day (humidity 49%, room temperature 26°C).

#### 2.2.2 POM on tibial cartilage

Six porcine knee joints (6 months old, ∼100 kg bodyweight) were obtained from a local butcher for friction experiments in the robotic tribometer. After removing the soft tissues, the femur and tibia were separated, and the menisci were detached from the tibial plateau using a scalpel. The tibial plateau was cut to a height of approximately 50 mm using a bone saw. The distal end was embedded in polymethylmethacrylate (Technovit 9100, Kulzer GmbH, Germany) to align the sample surface plane-parallel to the plate. Three holes were drilled into the embedding to be screwed on the sample plate. During sample preparation AC hydration was maintained with phosphate-buffered saline (PBS) (Gibco™, Thermo Fisher Scientific, Germany) and care was taken not to damage the cartilage surfaces on which the friction tests should be performed. A hemispherical POM pin (Ø 12.7 mm) was used as a friction partner. The workflow and friction testing protocol were as follows: First, a 3D model of the tibial plateau was generated using a structured-light 3D Scanner (Shining EinScan SP V2 Shining 3D, 36 frames per object scan, total scan time: approximately 5 min, single scan resolution: 0.05 mm). During scanning, the plate with the tibial plateau was equipped with two custom-made alignment tools (Figure 3B). This procedure ensured that the position of the tibial plateau’s 3D model in the virtual tribometer matched its position in the real-world tribometer. The AC remained without hydration during 3D scanning. To avoid any effect of dehydration on the frictional properties, the AC surface was covered with a PBS-soaked gauze for 30 min prior to testing. In the virtual tribometer, the 3D model of the tibial plateau was used to define three trajectories (inner, central, outer) on the medial and lateral AC surfaces, respectively (Figure 3B). The trajectories were directed from anterior to posterior. The stroke length of the trajectories was dependent on the location on the tibial surface (ranging between 30 and 57 mm). On each trajectory, a constant normal force of 5 N and a velocity of 1 mm/s were applied for five reciprocating cycles (which corresponded to a test time of between 6 and 10 min). At each direction change (start and end positions of the trajectory), PBS was sprayed on the cartilage surface to ensure lubricated contact conditions. PBS was chosen because it has been widely used as a lubricant in *ex vivo* friction tests (Rajankunte Mahadeshwara et al., 2024). Two structural conditions were tested to identify the sensitivity of the proposed tribological analyses. First, the medial and lateral cartilage surfaces were tested in a macroscopically intact condition (Figure 3B). Immediately after testing, a visual inspection was performed to check whether the tested trajectories displayed wear induced by the friction tests. Subsequently, a local cartilage defect (∼10 × 10 mm) was created on the anteromedial area of the plateau using sandpaper (grit size 120) to test whether it was possible to detect differences in friction due to changes in cartilage structure (Figure 3B). This mechanically induced cartilage damage has been shown to increase friction and has previously been used to simulate osteoarthritis-like changes (Tanaka et al., 2005). The lateral tibial surface was left intact as a control to investigate the effect of repeated testing on friction.

#### 2.2.3 Data analysis and statistics

Data analysis was performed using a custom-made MATLAB script. First, the force data from the high-resolution friction force sensor were synchronized with the position data of the pin along the trajectory. This was performed using the force feedback when the pin reached the sample surface, registered by both, the robot and the friction force sensors. Next, the reversal points of the robot motion were excluded from the analysis by deleting the data from the first and the final 2 mm of the trajectory. This was applied to avoid the effects of non-zero relative velocity at direction changes, which could influence friction. For every cycle, local friction coefficients were calculated at each position along the trajectory from the ratio of the friction force to the normal force (Desrochers et al., 2013; Pogačnik and Kalin, 2012). These local friction coefficients were then averaged over all cycles (µ_local_). The tool’s position data were used to plot µ_local_ along the trajectory. For statistical comparisons, the local friction coefficients of the trajectories were averaged, representing the mean friction coefficient of the trajectory (µ). Moreover, to evaluate whether a constant force application could be achieved with the robotic tribometer, the mean applied normal force was calculated for each trajectory. All statistical analyses were performed using a statistical software package (Prism 10, GraphPad Software Inc., United States). Shapiro-Wilk testing indicated normal distribution of the results. Therefore, the following statistical tests were applied.

• POM on PA: One-way analysis of variance with Tukey correction for multiple testing for differences in µ between the three PA sample shapes
• POM on tibial cartilage: Paired t-test for differences in µ between the intact and defect AC conditions

## 3 Results

### 3.1 POM on PA

**FIGURE 4:**
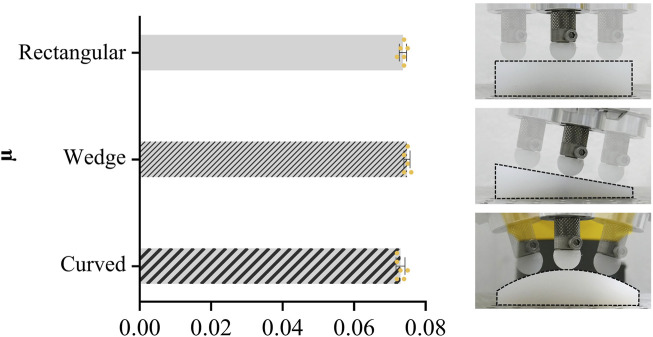
Mean ± SD values of the friction coefficient µ assessed in the POM-on-PA experiments with three different PA shapes (rectangular, wedge, curved); n = 6 trajectories.

The applied normal force ranged between 4.995 and 5.002 N (Table 1). The friction coefficients of the three PA shapes investigated were not statistically different (p > 0.05; Figure 4). The determined µ values ranged between 0.072 and 0.076. The highest mean value was found for the wedge-shaped geometry (µ = 0.075 ± 0.001), followed by the plate geometry (µ = 0.074 ± 0.001). The lowest friction was measured on the curved geometry (µ = 0.073 ± 0.001). Graphs presenting representative raw data of the applied normal and resulting friction force along a trajectory for each of the PA shapes are provided in the Supplementary Material (Supplementary Figure S1).

**TABLE 1:** Summary of the mean normal force applied during the POM-on-PA experiments.

	Rectangular	Wedge	Curved
Applied normal force in N Mean ± SD (n = 6)	5.002 ± 0.004	4.997 ± 0.003	4.995 ± 0.006

### 3.2 POM on tibial cartilage

**FIGURE 5:**
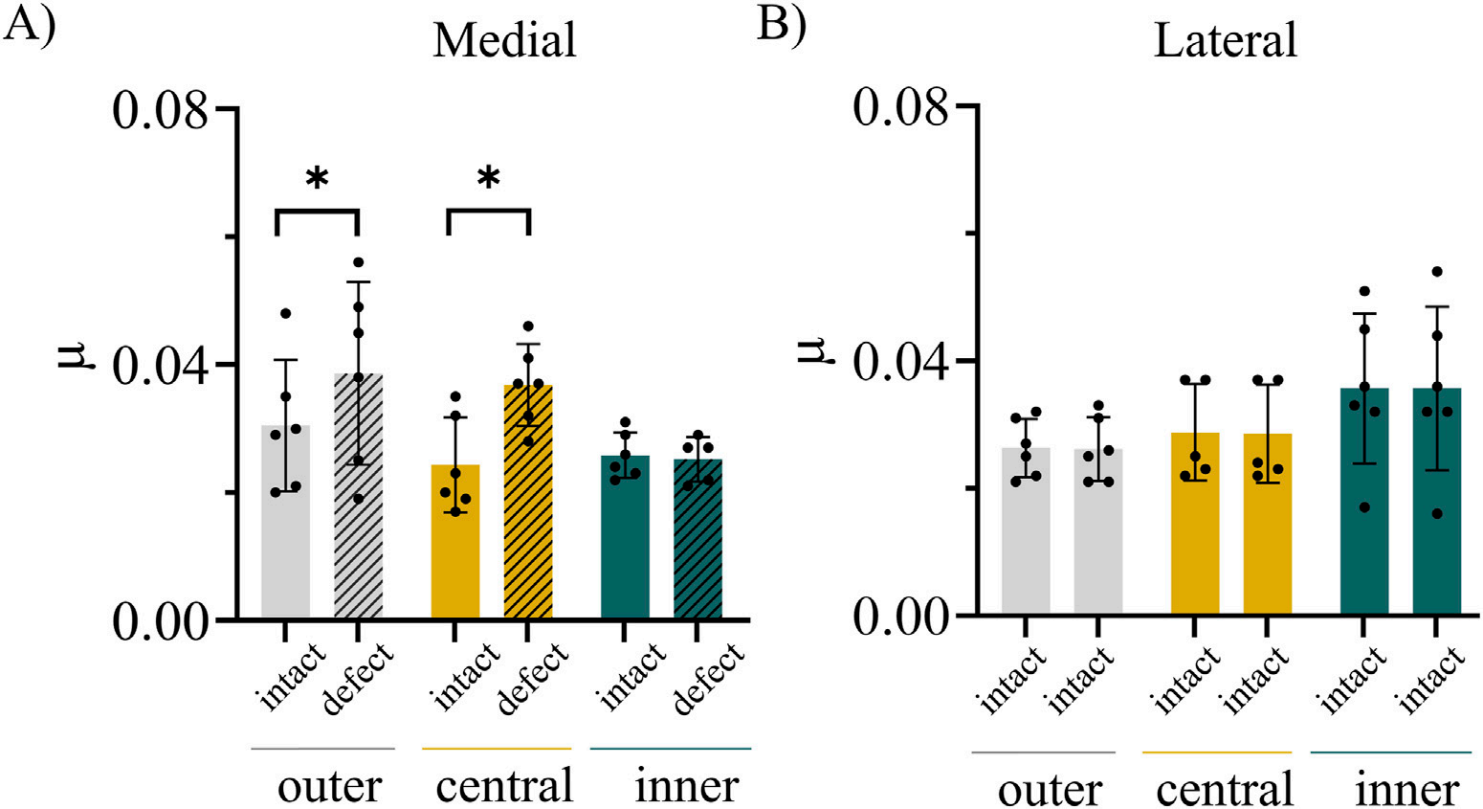
Mean ± SD values of the friction coefficient µ assessed on three trajectories (outer in grey, central in yellow, inner in green) on the medial **(A)** and lateral **(B)** articular cartilage surfaces of porcine tibial plateaus. Results of the intact cartilage conditions are represented as filled bars, those of the defect conditions as patterned bars. n = 6; *: p < 0.05, paired t-test.

**FIGURE 6:**
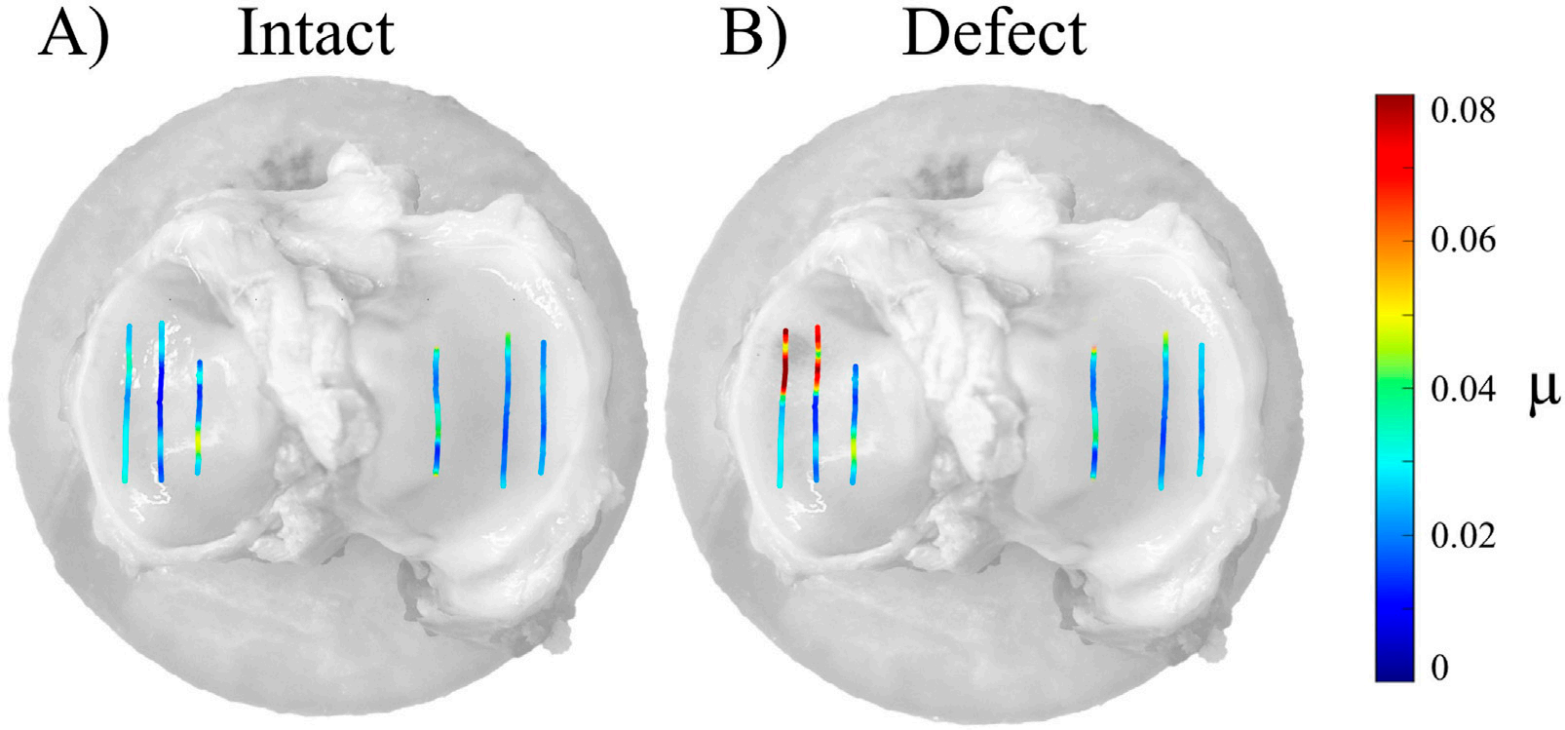
Representative visualization of the local friction coefficients µ_local_ on the six investigated trajectories (2D view in the x-y plane). Two articular cartilage conditions were investigated: **(A)** intact and **(B)** with anteromedial defect.

The mean normal force applied in the POM-on-tibial cartilage experiments ranged between 4.992 and 4.996 N (Table 2). The friction coefficients of the medial trajectories ranged between 0.022 and 0.041, those of the lateral trajectories indicated a range of 0.024–0.034 (Supplementary Table S1). Paired t-testing indicated significantly increased µ values after induction of an anteromedial AC defect compared to the intact AC conditions for the outer (p = 0.036) and central (p = 0.003) trajectories (Figure 5). By contrast, no significant difference was found between the intact and defect AC conditions for the inner medial trajectory (Figure 5). Visualization of the local friction coefficients along the trajectory of the intact and defect AC conditions demonstrated that the increase in friction observed for the outer and central medial trajectories was attributable to higher µ_local_ values in the defect area on the anterior portion of the medial plateau (Figure 6).

**TABLE 2:** Summary of the mean normal force applied in the POM-on-tibial cartilage experiments on the outer, central and inner trajectories on the medial and lateral surfaces.

		Outer		Central		Inner	
		Intact	Defect	Intact	Defect	Intact	Defect
Applied normal force in N Mean ± SD (n = 6)	Medial	4.995 ± 0.003	4.994 ± 0.006	4.994 ± 0.006	4.993 ± 0.003	4.995 ± 0.003	4.992 ± 0.003
	Lateral	4.996 ± 0.004	4.996 ± 0.005	4.996 ± 0.005	4.995 ± 0.004	4.988 ± 0.003	4.990 ± 0.006

## 4 Discussion

The aim of this proof-of-concept study was to demonstrate the suitability of a newly developed tribometer that allows for the investigation of region-specific AC friction according to Coulomb’s law while preserving the complex anatomy of the tissue and thus its integrity. First, the results of the POM-on-PA experiments revealed that the complexity of the surface geometry did not influence the measured friction coefficients, because no differences in µ were found between the rectangular, wedge and curved PA samples. Consequently, the first hypothesis of the study was confirmed. Second, we demonstrated that the robotic tribometer is able to detect differences in AC friction due to structural tissue damage, thus confirming the second hypothesis of the study.

### 4.1 POM on PA

The robotic tribometer was considered to have been successfully validated because the friction coefficients of the POM-on-PA experiments showed no statistically significant differences between the three PA samples of different shape complexity. When comparing the results with literature values, the present friction coefficients were lower than those previously reported, which ranged between 0.2 and 0.5 (Pogačnik and Kalin, 2012). This is most likely related to differences in the test conditions. In previous tribological studies on this material pairing, considerably higher velocities (500–1,000 mm/s) and normal loads (50–500 N) were applied (Rajankunte Mahadeshwara et al., 2024; Miler et al., 2019; Pogačnik and Kalin, 2012), because its final application in, for example, gears must withstand such operational conditions (Miler et al., 2019). By contrast, we used a much lower velocity (1 mm/s) and normal load (5 N) in our POM-on-PA experiments. The rationale was that we aimed to validate the system’s performance under conditions relevant for the AC experiments. Sliding velocities identified as physiologically reasonable for biotribological studies ranged between 0.5 and 100 mm/s (Rajankunte Mahadeshwara et al., 2024; Warnecke et al., 2019; Burris and Moore, 2017; Burris et al., 2019; Neu et al., 2010). Normal forces are usually selected to achieve a contact pressure between 0.1 and 0.9 MPa (Rajankunte Mahadeshwara et al., 2024; Warnecke et al., 2019; Burris and Moore, 2017; Burris et al., 2019; Neu et al., 2010). We applied a velocity of 1 mm/s and normal force of 5 N in our experiments, because cartilage friction is well-documented for these test parameters (Warnecke et al., 2019). For thermoplastic materials like POM and PA, their friction coefficients have been demonstrated to increase in both, a velocity- and load-dependent manner (Pogačnik and Kalin, 2012). This may be the reason for the lower friction coefficients observed in the present study in comparison with those reported in the literature.

### 4.2 POM on tibial cartilage

The friction coefficients determined on the intact AC ranged between 0.017 and 0.031, which is comparable to previously reported literature values for healthy animal AC assessed in pin-on-plate tests (Warnecke et al., 2019; Schütte et al., 2020; Schwarz et al., 2022). Roughening the AC at the anteromedial region of the tibial plateau with sandpaper resulted in a 30%–50% higher friction coefficient of the outer and central trajectories. On both trajectories, the anteromedial portion was affected by the mechanically induced surface roughening. The visualization of the local friction coefficients along the trajectory clearly demonstrated that the overall increase in friction was attributable to increased µ_local_ values in the defect area. Here, the local friction coefficients displayed approximately 2.5-fold higher values compared to those at the unaffected positions along the trajectory (∼0.08 vs. ∼ 0.03, Figure 6). This is in agreement with previous passive pendulum studies in which sandpaper was used to model osteoarthritic cartilage damage in the porcine hip (Mori et al., 2002) and temporomandibular joint (Tanaka et al., 2005). In both studies, the mechanically induced cartilage damage resulted in higher whole joint friction compared to the intact joint conditions, with respective values of 137% (Mori et al., 2002) and 247% (Tanaka et al., 2005). Compared to these passive pendulum studies, our method offered more detailed insights into local differences. In our experiments, no statistically significant increase in µ was observed on those trajectories on which the AC condition was left intact during the second test run. This was the case for the inner medial trajectory and all lateral trajectories. From these results, it can be concluded that multiple testing did not affect the measured friction coefficient. This was consistent with the visual inspection of the AC surface on these trajectories after friction testing, where no macroscopic AC damage was observed.

### 4.3 Limitations and challenges

This proof-of-principle study has limitations that need addressing. Although the robotic tribometer was designed for friction analysis of biological tissues, we chose thermoplastics for validation purposes, which do not exhibit the same tribological characteristics as AC (Bonnevie and Bonassar, 2020). Despite this, investigating this material pairing allowed validation without any effect of biological variability. We assessed the friction coefficients of POM-on-PA under dry sliding conditions rather than in a lubricated environment for two reasons. First, the dry frictional properties of this material pairing are well described in the literature (Rajankunte Mahadeshwara et al., 2024; Miler et al., 2019; Pogačnik and Kalin, 2012). Second, we decided to exclude the complexity of lubrication in these experiments, because we were primarily interested in frictional differences due to the sample geometry. Although lubrication would probably have resulted in even lower friction coefficients, the values presented are in the middle of the range reported for AC (Rajankunte Mahadeshwara et al., 2024). Therefore, we concluded that the robotic tribometer was suitable for investigating AC friction. The aforementioned biological variability was also the rational for using a nonpermeable pin made of POM in the friction experiments on the tibial plateaus, although this was a non-physiologic material pairing. Using a standardized pin material in the proof-of-concept experiments allowed for the tribological characterization without the involvement of a friction partner, which itself is characterized with a complex tribological behavior (Ruggiero, 2020; Jahn et al., 2016). Following the herein presented experiments, upcoming studies with the robotic tribometer might focus on physiologic material pairings by using meniscus or AC samples as the pin. Furthermore, it was not possible to determine the actual contact area and thus the actual contact pressure between the hemispherical pin and the AC along the trajectory. Under a constant normal load, the contact area and, therefore, the contact pressure depends predominantly on the local stiffness of the AC. In both the POM-on-PA and POM-on-tibial cartilage experiments, the applied normal force showed minimal deviation from the target value. This indicated that the robot’s controlled force application was suitable to maintain a constant normal force regardless of the material pairing. However, in the POM-on-tibial cartilage experiments, it is likely that the contact area and pressure differed along the trajectories, because cartilage stiffness is known to vary throughout the tibial surface (Seitz et al., 2021). On the basis of the well-documented load dependence of cartilage friction (Furmann et al., 2020; Gleghorn and Bonassar, 2008), it would be valuable to establish a method to assess the actual contact area, taking into account Hertzian contact mechanics, in order to consider local contact pressures during testing. Trajectory planning was performed with reference to the surface of the sample’s 3D model in the virtual tribometer. Therefore, an exact representation of the real sample in the virtual tribometer is crucial. While the digital representation of the PA shapes was readily available from the CAD process, creation of a 3D model of the tibial plateaus was required. To generate accurate digital twins, a 3D scanner with a high-resolution of 0.05 mm was used.

In this study, we investigated the frictional properties using a single normal load and testing velocity. The test duration of each trajectory was approximately 10 min. Therefore, we did not investigate long-term friction. We chose the applied test parameters because the normal load and testing velocity were those used in previous tribological tests on cartilage tissue (Warnecke et al., 2019; Warnecke et al., 2017), therefore, enhancing comparability with the literature. While the investigation of the dependence of friction on test conditions or testing duration was beyond the scope of this proof-of-concept study, the technical specifications of the robot system do allow for future investigations under various and more challenging loading conditions. These include, for example, experiments under loading conditions derived from the stance phase of gait with a high normal load and low velocity and the swing phase of gait with a low normal load and high velocities (Warnecke et al., 2019).

## 5 Conclusion

The presented proof-of-concept study demonstrated that the robotic tribometer is suitable for the investigation of friction on curved joint surfaces while maintaining AC integrity. Moreover, it was possible to detect local differences in AC friction attributable to structural changes. This is of particular interest in the context of osteoarthritis, where the affected articular joint surfaces display distinct localized AC wear patterns (Van Oevelen et al., 2022). Whether this is related to locally increased friction remains unclear, partly because the methods used to date lack in local differentiation of the results (pendulum) or require the extraction of the tissue out of its natural environment (pin-on-tribometer). The robotic tribometer now offers a novel methodology to gain new insights into the complex tribology of AC tissue with respect to its structural integrity. Overall, this has the potential to advance our understanding of the knee joint’s friction-related functionality in health and disease.

## Data Availability

The raw data supporting the conclusions of this article will be made available by the authors, without undue reservation.
