# Supplementary material for: Proof-of-concept study of the TriBot: a robot-based test setup for biotribological analyses of curved articular surfaces
Source: Front Bioeng Biotechnol. 2025 Apr 25;13:1546060. doi: 10.3389/fbioe.2025.1546060 (PMC12062063; doi:10.3389/fbioe.2025.1546060)
Supplement: Supplementary file 1 [file DataSheet1.pdf]

Supplementary Material

1 Supplementary Figures and Tables

1.1 Supplementary Figures

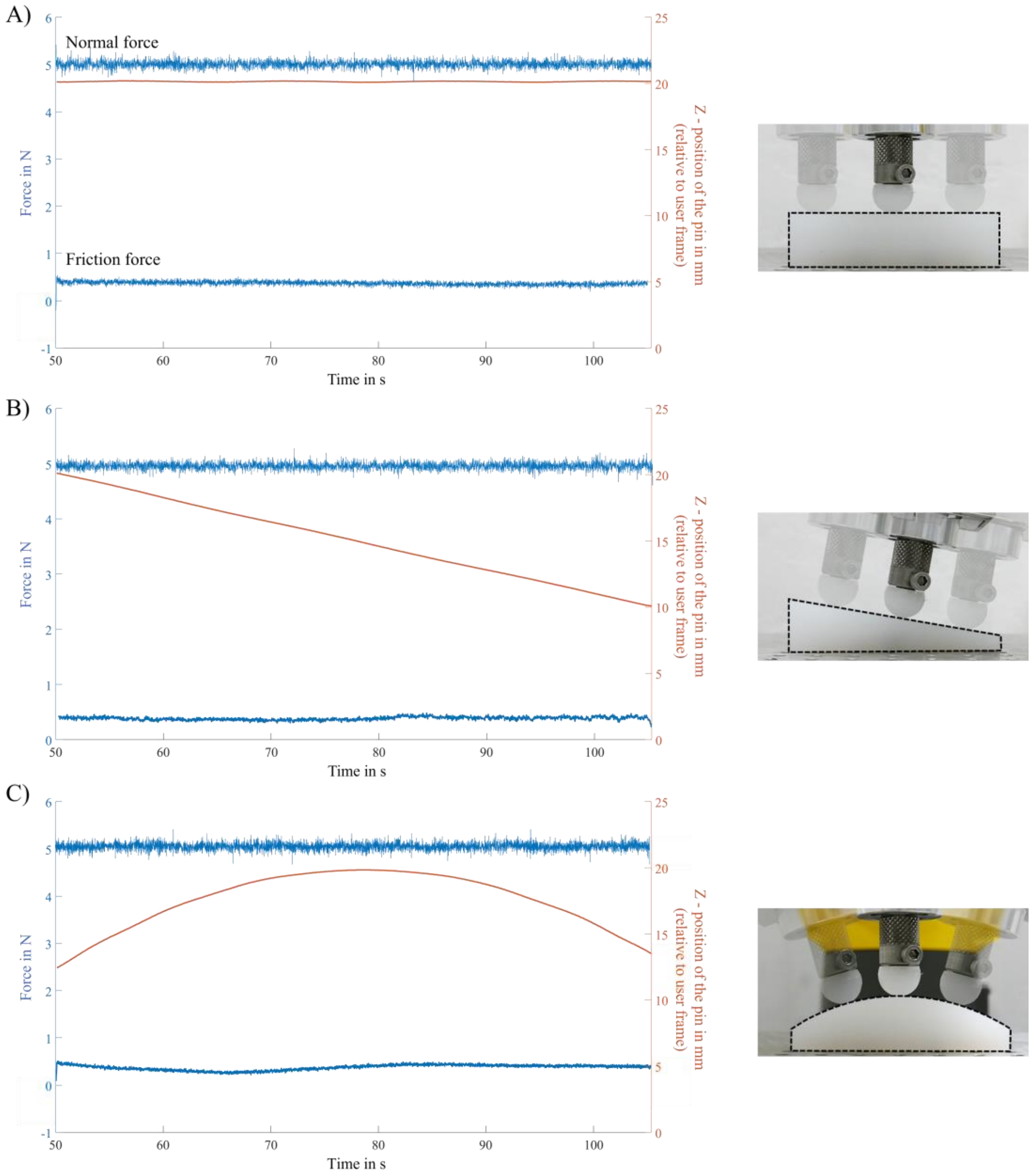

Supplementary Figure 1. Exemplary raw data recorded along a trajectory on the three different PA shapes A) rectangular B) wedge and C) curved. On the left y-axis of each graph (blue), the applied normal force (target value of 5 N) and the resulting friction force in N are shown. On the right y-axis (orange), the corresponding z-position of the pin relative to the user frame is displayed in orange over time.

1.2 Supplementary Tables

Supplementary Table 1: Summary of the mean friction coefficients of the POM-on-tibial cartilage experiments on the outer, central and inner trajectories on the medial and lateral surfaces.

		Outer		Central		Inner	
		Intact	Defect	Intact	Defect	Intact	Defect
Friction coefficient Mean ± SD (n = 6)	Medial	0.031 ± 0.010	0.039 ± 0.014	0.024 ± 0.007	0.037 ± 0.006	0.026 ± 0.004	0.025 ± 0.003
	Lateral	0.026 ± 0.005	0.026± 0.005	0.028 ± 0.008	0.029± 0.008	0.036 ± 0.012	0.036 ± 0.013
